# Physician-scientist development: a new category for supporting all stages of the physician-scientist pipeline

**DOI:** 10.1172/jci.insight.179940

**Published:** 2024-03-08

**Authors:** Kathleen L. Collins

One of the major focuses of the American Society for Clinical Investigation (ASCI) is support for physician-scientists at all stages of their careers. In accordance, the ASCI has initiated multiple programs to provide support and mentorship to help maintain a robust pipeline of physician-scientists. The success of combined MD-PhD graduates nationally in obtaining faculty appointments at outstanding medical schools and in obtaining NIH funding attests to their importance at the basic science-clinical interface. Although MD-PhD students constitute only 3% of medical school graduates, they are almost 50% of the NIH-funded physician-scientists (1). This success has been documented in the Association of American Medical Colleges 2018 National MD-PhD Program Outcomes Study (2). However, this study also noted that the “number of [MD-PhD] graduates is just over half the number that the Physician Scientist Workforce (PSW) report estimated will be needed to sustain the workforce at current levels” (1), implying a need for increased numbers of physician-scientists from a variety of different pathways. Various challenges faced may hinder matriculation through physician-scientist training programs and postdegree advancement, resulting in an unstable physician-scientist workforce. For example, the training is complex and lengthy, and combined degree programs receive relatively low numbers of applications from underrepresented groups (3). These and other challenges threaten the success of crucial physician-scientist pipelines. A better understanding of issues that hinder recruitment, advancement, and maintenance of physician-scientists in the pipeline is essential for implementing changes to reduce these hurdles, especially for those underrepresented in medicine and science, and ensuring a robust future for clinician-led research programs.

In support of this mission of the ASCI, *JCI Insight* is pleased to announce the launch of the Physician-Scientist Development category. This new category will support publications of studies focused on approaches that will improve physician-scientist education, training, retention, and career development. We are particularly interested in studies that include quantitative metrics, outcomes data, and actionable recommendations. Since the launch of *JCI Insight* in 2016, we have been honored to publish a limited number of invited Perspective articles (https://insight.jci.org/tags/53) that address physician-scientist–specific issues, such as funding success, increased training time, gender parity in admissions, and a recent article looking at how sociodemographic factors and research experience affect acceptance into MD-PhD programs (4). In this issue, we feature an article in our newly formalized Physician-Scientist Development category (https://insight.jci.org/collections/topic/articles) that describes workshops developed at Yale University that provide formal instruction during the development and submission of predoctoral fellowship applications and the effect of this program on fellowship funding success (5).

With the launch of this category, authors will now be able to directly submit their studies on Physician-Scientist Development directly to *JCI Insight* using our submission platform. We look forward to your submissions.

## Version 1 03/08/2024

Electronic publication

## Version 2 03/18/2024

A link to the Physician-Scientist Development collection was added
